# Reassuring and managing patients with concerns about swine flu: Qualitative interviews with callers to NHS Direct

**DOI:** 10.1186/1471-2458-10-451

**Published:** 2010-08-02

**Authors:** G James Rubin, Richard Amlôt, Holly Carter, Shirley Large, Simon Wessely, Lisa Page

**Affiliations:** 1 King's College London, Institute of Psychiatry, Department of Psychological Medicine, Weston Education Centre (PO62), Cutcombe Road, London SE5 9RJ, UK; 2 Health Protection Agency, Emergency Response Department, Porton Down, Salisbury, Wiltshire, SP4 0JG, UK; 3 NHS Direct, Strawberry Fields, Berrywood Business Village, Tollbar Way, Hedge End, Hampshire, SO30 2UN, UK

## Abstract

**Background:**

During the early stages of the 2009 swine flu (influenza H1N1) outbreak, the large majority of patients who contacted the health services about the illness did not have it. In the UK, the NHS Direct telephone service was used by many of these patients. We used qualitative interviews to identify the main reasons why people approached NHS Direct with concerns about swine flu and to identify aspects of their contact which were reassuring, using a framework approach.

**Methods:**

33 patients participated in semi-structured interviews. All patients had telephoned NHS Direct between 11 and 14 May with concerns about swine flu and had been assessed as being unlikely to have the illness.

**Results:**

Reasons for seeking advice about swine flu included: the presence of unexpectedly severe flu-like symptoms; uncertainties about how one can catch swine flu; concern about giving it to others; pressure from friends or employers; and seeking 'peace of mind.' Most participants found speaking to NHS Direct reassuring or useful. Helpful aspects included: having swine flu ruled out; receiving an alternative explanation for symptoms; clarification on how swine flu is transmitted; and the perceived credibility of NHS Direct. No-one reported anything that had increased their anxiety and only one participant subsequently sought additional advice about swine flu from elsewhere.

**Conclusions:**

Future major incidents involving other forms of chemical, biological or radiological hazards may also cause large numbers of unexposed people to seek health advice. Our data suggest that providing telephone triage and information is helpful in such instances, particularly where advice can be given via a trusted, pre-existing service.

## Background

The 2009 outbreak of influenza A (H1N1), or 'swine flu,' killed 457 people in the UK between April 2009 and March 2010 [1]. Although the illness turned out to be relatively mild for most people, the early stages of the outbreak were characterised by uncertainty as to the nature and severity of the illness, with concern being expressed about the ability of medical services to cope with the volume of patients who might require help. While most attention focused on the ability of hospitals and primary care trusts to care for patients suffering from swine flu, [2] in practice most people who initially sought help did not have the illness. In the UK, NHS Direct, which is a 24 hour telephone and online health advice and information service staffed primarily by nurses and health information advisors, received approximately 63 000 calls relating to swine flu in the first month of the outbreak [3]. During the same period only 185 cases of swine flu were confirmed across the UK [4]. Although the tendency for large numbers of unaffected individuals to seek advice and assistance from the health services is a common occurrence during major public health incidents, [5,6] little evidence exists as to what factors motivate these patients to seek help. A common assumption is that this group consists of worried patients who require reassurance. [e.g. [7,8]]

The role of NHS Direct in providing information and reassurance to concerned members of the public during the swine flu outbreak sat within a broader strategy designed to relieve pressure on primary care physicians. After the World Health Organisation declared on 29 April that a pandemic was imminent, widespread Government advertisements about the condition began to appear in the UK, including an information leaflet delivered to every house in the country [9]. These mass media campaigns were supplemented by websites and an automated telephone line which contained more detailed information. Members of the public were advised that if they had "just returned from Mexico or an affected country" and "think [you] might have swine flu" they should check their symptoms on-line, call the automated telephone number and then telephone either their GP or NHS Direct "if you have taken these steps and are still concerned." [9] Similar remote triage systems have also been used in other countries during both the swine flu outbreak and in previous major public health incidents [10,11]. Similar systems are also recommended for future incidents [5,6].

While patients typically report that NHS Direct is a helpful and reassuring source of information and advice during normal circumstances, [12] whether this holds true during a major public health incident is less certain. Such incidents are often characterised by high levels of uncertainty about the severity of the risk that people face, uncertainty as to who is most at risk, the presence of conflicting advice from experts and official agencies, and extensive media coverage and speculation. All of these issues tend to increase levels of public concern [13] and make it difficult to provide effective reassurance to patients. Indeed, during one previous major incident in the UK, some people who spoke to NHS Direct or to the UK's Health Protection Agency by telephone reported that this contact added to their confusion or made them suspicious as to why they had been asked to contact these services at all [14].

In this study we used qualitative interviews with patients who contacted NHS Direct about swine flu during the early stages of the outbreak in order to assess whether the advice and reassurance given to them adequately met their needs and to explore the factors that were most reassuring for patients. Because we were also interested in understanding why people contacted the health services about swine flu during this period, despite most not having been exposed to the illness, we also asked a range of questions relating to patients' motivations for seeking advice.

## Methods

### Participants

People were eligible for this study if they had contacted NHS Direct between 11 and 14 May 2009 in order to discuss swine flu, were 18 years or older, spoke English and had received only information or treatment at home advice. Our intention was to interview 30 to 40 patients who met these criteria. From prior experience [14] we felt that this number would allow us to identify the most important reasons people had for making contact with NHS Direct.

### Procedure

One NHS Direct call centre participated in the study. Call centre staff assessed eligibility and requested consent for contact details to be passed to us. Staff members were asked to recruit up to five callers each. The interviewers (RA, HC, GJR and LP) called consenting patients and informed them about the purposes of the research. Participants were again asked to verbally confirm their consent to take part in the interviews and were informed that all data would be anonymised by the removal of any personally identifiable material from our transcripts and publications. Participants were then guided through a semi-structured interview lasting 10 to 15 minutes (see Appendix 1). The questions asked in these interviews were specifically chosen in order to explore several key topics of interest that were identified a priori. We also asked one item adapted from the state trait anxiety inventory: "when you called NHS Direct, how worried were you about the swine flu outbreak?" [15] Permitted responses were 'very much,' 'moderately,' 'somewhat' and 'not at all.'

### Analyses

Interviews were digitally recorded and transcribed. Qualitative analysis was conducted using a technique akin to a framework approach, [16] in that we approached the data with a set of pre-determined main themes in mind which we wished to explore. These were: reasons for wanting to speak to someone about swine flu; reasons why any prior information sources had not been sufficient; reasons motivating patients to speak to NHS Direct rather than to a different healthcare provider; reasons why speaking to NHS Direct had been reassuring; reasons why speaking to NHS Direct had provoked concern or anxiety; and reasons for wanting to seek more advice about swine flu after speaking to NHS Direct. We first looked for quotes from each transcript which related to one of these main themes. Within each theme, we then grouped quotes together which appeared to reflect the same underlying sub-theme, using an iterative process of refining and re-labelling these sub-themes. Once themes were identified, two researchers re-examined each transcript and identified how many participants had explicitly mentioned each sub-theme to ensure that they reflected general experiences among our participants rather than idiosyncratic findings relating to single people.

### Ethics

The National Research Ethics Service advised us to treat this study as a service evaluation, exempt from research ethics approval.

## Results

Between 11 and 14 May, the call centre received 583 calls concerning swine flu, of which 349 received home care advice and 40 received information only. 45 patients gave permission for their details to be passed to us. Of these, we interviewed 33. 32 participants were interviewed within two weeks of their contact with NHS Direct; the remaining participant was interviewed after 16 days.

Our participants included 19 women (58%), had a median age of 33 years (range: 19 to 79), and were predominantly white British (n = 24, 73%), with the remainder being 'mixed White' (4, 12%), Indian (2, 6%) and 'other Asian,' Black African or Turkish Cypriot (1 each, 3%). Three participants had called NHS Direct on behalf of a relative.

### Levels of worry before calling NHS Direct

Fifteen participants (45%) reported that when they had called NHS Direct they were 'not at all' worried about swine flu. Eight were 'somewhat' worried, seven were 'moderately' worried and three were worried 'very much.'

### Qualitative results

Appendix 2 summarises the sub-themes that emerged within each of our main themes.

### Reasons for seeking advice about swine flu

Only two participants were asymptomatic when they initially sought information about swine flu. For them, the main motivating factor for seeking advice was a desire to discuss prophylaxis.

The remaining 31 participants had experienced flu-like symptoms or were phoning on behalf of someone who had. Within this group, the unusualness of their symptoms was often described as an important factor motivating them to seek advice, with unusualness typically defined in terms of unexpected severity or duration. For example, one participant noted that "I've had a cold and I've had the flu and it only lasted about 2 days. But I had this about a week and a half. It's the first time I've had a cold that long and I was just thinking the worst." Some participants also mentioned that they wanted information about any distinguishing characteristics of swine flu, particularly in terms of its symptoms.

Concern or uncertainty about having been exposed to swine flu was an additional factor motivating some participants to seek advice. Their explanations suggested a variety of perceptions as to what might count as exposure, ranging from mixing with others who had returned from an affected region ("I'm a teacher, and a number of the kids had come back from the States, so I thought it was a possibility"), to having been in roughly the same location as a known case ("I came down with flu symptoms and I work at [an] airport. And there was two confirmed cases of swine flu gone through"), to being among unknown people ("I'm in a [waiting room] where patients are coming in all the time. Nobody is walking round with a label or a badge on saying 'I've just been to Mexico'"). In contrast, other participants sought advice due to concern that they might expose others. For example, one commented that "I work with children all day, so it's more for my fear that if I've got something like that, then it would go amongst children," while another explained that "I just phoned up to make sure that I didn't have [swine flu]..., because I was visiting my parents and they're quite elderly and I was a bit worried."

Pressure from others was important for many participants. For some, this pressure came from friends or family. For others, it came from their employer and was not always well-received (e.g. "I kind of thought I just had a bug. My school wouldn't let me go back until I'd had it confirmed that I didn't have swine flu... I thought it was quite a ridiculous assumption").

For some patients, another motivation for seeking advice was a desire to obtain "peace of mind," "confirmation," or "reassurance" that they did not have swine flu. These participants appeared to recognise that they were unlikely to have it, but felt that they should err on the side of caution nonetheless (e.g. "[I wanted] to confirm that I hadn't got this flu virus. So it was more so, you know, 'you *definitely *haven't.' That was the reassurance I needed").

Finally, some participants mentioned that personal risk factors had increased their motivation to speak to someone, for example age or pre-existing chronic illness.

### Information sources tried before calling NHS Direct

Before telephoning NHS Direct, participants had variously sought information from the NHS Direct website, the Government leaflet on swine flu, their GP, other official websites, a pharmacy and a hospital. Three themes emerged as to why people felt they needed additional advice despite having already used these sources. First, a common reason was simply because the participant had been explicitly told that they needed to seek help from elsewhere. Second, people who had used an internet resource or the Government leaflet also made comments suggesting that they now wanted to speak to a human, because "you can read so much in a leaflet, and you can watch so much, but I think when you speak to someone it's a bit more reassuring." Finally, a lack of trust in the initial source was important for some participants.

### Why NHS Direct?

Five themes reflected why participants eventually called NHS Direct, rather than seeking advice elsewhere. For some participants, it was because they had been informed that NHS Direct was the most appropriate place to get help, while others commented that using a telephone service would reduce their chances of giving swine flu to friends, family members or colleagues. Also important was the ease with which NHS Direct could be accessed. As one participant noted "it's a lot less hassle than going to your GP." Trust in NHS Direct was mentioned by many participants. This often related to a previous positive experience with them (e.g. "any time I ring NHS Direct about anything, it's always reassuring"). Finally, some participants mentioned that they wanted to avoid burdening other medical services.

### Reasons why speaking to NHS Direct had been reassuring

Most participants did obtain reassurance from speaking to NHS Direct. Although some indicated that speaking to NHS Direct had not been reassuring, in each instance this reflected the fact that the participant had not been worried to begin with. One factor underlying the reassurance was the ability of staff to explicitly rule out swine flu. In addition, providing an alternative explanation for symptoms was mentioned as reassuring (e.g. "I wanted to check that there were just other kinds of flu bugs going around, because it surprised me that at this time of year I should be having these sort of symptoms. So really I wanted somebody to say yes there are, they do last a few days, it's all perfectly normal").

Helping patients to understand that they were only at risk of catching swine flu if they had been in close contact with a known case was also reported as reassuring. For example, one participant was reassured after finding out that "you definitely need to have had contact for a sustained period, i.e. over an hour," while another remembered being told that "nobody in [my area] had been confirmed as contracting swine flu, so therefore the likelihood [that I had caught it] was probably extremely small." For other participants, it appeared that confusion still existed about their potential for having been exposed. For some, this emerged as only a minor nagging doubt. For others, it was a more central issue which diminished the reassurance that they had obtained. For example, one participant was unhappy that she had been asked if her family member had come into contact with a known case and commented that; "well you don't know, that's the thing. They were very unhelpful... It just wasn't reassuring... They say you don't fit the criteria, but according to that list [of symptoms], you know, everything that she had is on that list."

While factual aspects of their call relating to diagnoses or exposure were helpful for many patients, less tangible aspects relating to the credibility, expertise or professionalism of NHS Direct were also reported as reassuring. This was suggested by comments such as "[I was] talking to someone who knew what they were talking about," "it wasn't just a quick chat [but] I didn't feel like I was wasting their time," "they were quite, what's the word, quite warm" and "[she] was the first person I spoke to that I felt I had any confidence in." Related to this, the simple action of telling the patient that they could call back if they had any additional concerns was explicitly cited as reassuring by some participants.

### Did speaking to NHS Direct provoke concern or worry?

None of the participants were able to identify any aspect of their call that had caused them concern or worry.

### Use of other medical services following contact with NHS Direct

Only one participant reported seeking additional advice about swine flu after speaking to NHS Direct. The employer of this participant had asked her to re-check the cause of her symptoms. She did this by speaking to NHS Direct again and by making an appointment with her GP.

## Discussion

Incidents involving the release of a chemical, biological or radiological hazard often result in a large number of unexposed people seeking medical advice [6,7,17-20]. The early stages of the swine flu outbreak was no exception [3]. While previous reports have highlighted the need for strategies that are designed to reduce the number of unexposed patients who seek care in future incidents, surprisingly little is known about the factors which produce this 'surge' of patients [5,6] A common perception has previously been that such patients are "the worried well." [e.g. [7,8]] However, our results suggest that this is inaccurate. For our participants, the presence of worry or a desire for 'peace of mind' were only some of the motivations that were cited for seeking advice: worry was by no means a necessary prerequisite for someone making contact with NHS Direct. By identifying other factors underlying health care use during a major incident we may be able to both reduce the extent to which patients use these services unnecessarily and alter services to ensure that the needs of this patient group are met.

Other reasons for making contact that were identified in this study included a desire for information about prophylaxis and a need to understand how swine flu is transmitted. Partly, these motivations appeared to reflect the perceptions about swine flu that were held by our participants. While a large amount of research has demonstrated that how patients with medical conditions react to their illnesses largely depends on their perception of factors such as the cause, consequences or controllability of their illness, [21] recent evidence also suggests that how healthy people perceive a given illness can determine their desire to engage in behaviours that are intended to prevent them from becoming ill [22]. For example, research by our team has shown that perceptions regarding the infectivity of an emerging infectious disease are a strong predictor of intentions to seek medical advice during a hypothetical outbreak of that disease [23]. Additional research to clarify which perceptions about a major public health incident or a novel hazard are most important in determining how people respond to it is ongoing.

As with previous incidents [14] external pressure from friends, family members and employers was another key reason cited by patients for contacting NHS Direct. While it may be difficult in future incidents to prevent concerned friends and family members from encouraging unexposed individuals to make contact with the health services, providing improved information to employers may be possible. Where relevant, assuring employers that their staff do not require special certification in order to return to work may help to reduce the number of individuals who are encouraged to make contact with health services.

Understanding motivations for health care use is a useful first step in providing unexposed yet concerned patients with appropriate care. In this respect, several aspects of contact with NHS Direct were singled out as particularly useful or reassuring. As might be expected, advice that specifically related to swine flu and the evolving situation was important. But other aspects of the call which did not directly relate to swine flu were also important. In particular, the ability of NHS Direct staff to provide an alternative explanation for symptoms was reassuring for many participants, while elements of the conversation such as the warmth, professionalism or credibility of the staff member were also important. These latter themes, which have been identified before in surveys of NHS Direct users, [12] also helped motivate people to use the service in the first place. While capacity issues may sometimes require the setting up of a new specialist helpline following a major incident, using established services with which people have already formed a relationship and which can advise on a broad range of healthcare topics may be preferable.

### Methodological limitations

Given the high workload of NHS Direct staff during our study, the most pragmatic way to obtain sufficient participants for our analysis was to ask call centre staff to recruit up to five participants each. This essentially provided us with a convenience sample. As such, we cannot provide any meaningful response rate for the study: it is not clear how many participants were asked to participate in the research, but declined. Nonetheless, we believe that the themes we identified do reflect the key variables that were important in causing people to seek advice from NHS Direct and to find their contact with NHS Direct reassuring or useful. We have no reason to believe that our sampling excluded a large subset of patients whose experiences or perceptions were qualitatively different from those who were included. Although we were unable to feed back our results to the initial callers in order to gauge their credibility, presentation of our results to an NHS Direct call centre team suggested that the issues we identified did appear to be complete, although it is possible that some small subgroups, such as pregnant women or GPs may have had different concerns when they called in.

The relative importance of each of the subthemes that we identified is less clear. Quantitative research with a more robust sampling strategy would be required to answer questions such as what proportion of patients contact the health services due to worry, what proportion obtain benefit from a discussion of how one can become exposed to a particular hazard, and what proportion remain concerned about a major public health incident despite receiving reassurance.

Whether the sub-themes we identified are more generally applicable to other major incidents involving the release of a chemical, biological or radiological hazard, or whether they are specific to swine flu is also unclear. However, we have previously noted similar themes among members of the public who spoke to the Health Protection Agency following the dispersal of radioactive polonium 210 in central London in 2006 [14]. Additional research to identify the factors that motivate people to contact the health services during future incidents and the key aspects of that contact that help to reassure them is required.

## Conclusions

People who contacted NHS Direct about swine flu in the early stages of the outbreak had multiple reasons for doing so. Not all were anxious, while the majority of those who were gained reassurance from clear, factual information conveyed by a trusted source. For future major incidents in the UK, allowing existing services to provide initial telephone triage, advice and reassurance has much to recommend it.

## Competing interests

SL is a full-time employee of NHS Direct. She played no role in data collection or in the initial analysis of the data. The authors declare that they have no other competing interests.

## Authors' contributions

GJR had the original idea for the study and developed the study design with RA, LP, HC, SL and SW. Preliminary analyses were conducted by GJR, who also wrote the first draft of the paper. All authors contributed to further drafts, had full access to all of the data, and read and approved the final manuscript.

## Appendix 1. Open-ended questions used during our interviews with callers to NHS Direct. Interviewers were instructed to prompt for further detail as necessary

• Why did you call NHS Direct?

• Did you think that you, or a friend or relative, might have been exposed to swine flu?

• What were you hoping to get out of your call?

• Why did you contact NHS Direct rather than, say, your GP or local hospital?

• Before you called NHS Direct, had you tried to get advice or information from anywhere else about swine flu? Where had you tried? How useful did you find that?

• When you spoke to NHS Direct, what did they tell you?

• Did you find that reassuring or useful?

• What was the most reassuring part about your contact with NHS Direct?

• Did anything about your contact with NHS Direct make you concerned or worried?

• Is there anything that NHS Direct could have said or done that would have helped you more?

• Do you still have any unanswered questions about swine flu?

• Since you called NHS Direct, have you got more information about swine flu from anywhere else?

• Is there anything else you think we should pass on to NHS Direct that might help them to improve their service?

## Appendix 2: Sub-themes which emerged following a qualitative analysis of interviews with 33 patients who called NHS Direct about swine flu, but who probably did not have swine flu

### Reasons for seeking advice about swine flu

• Desire to discuss prophylaxis

• Experiencing unusual symptoms

• Seeking information about distinctive characteristics of swine flu

• Concern or uncertainty about exposure to swine flu

• Concern about exposing others to swine flu

• Pressure from others

• Peace of mind, confirmation or reassurance that patient does not have swine flu

• Risk factors such as having a young child or a medical comorbidity

### Reasons why any initial source of information was not sufficient

• Participant was told to seek help elsewhere

• Participant wanted to speak to a human

• Participant lacked trust in the initial source

### Reasons why the participant called NHS Direct

• Participant was told that NHS Direct was the appropriate place to seek help

• It lessens the chance of spreading the infection compared to going elsewhere

• Ease of access

• Participant trusted NHS Direct more than other sources

• Participant did not want to tie up other medical resources

### Reasons why speaking to NHS Direct was reassuring

• Ruling out swine flu-based on answers to the patient's questions

• Providing an alternative explanation for symptoms

• Clarifying the nature of 'exposure'

• The perceived credibility, expertise or professionalism of NHS Direct

• Being offered the chance to call back if necessary

## Pre-publication history

The pre-publication history for this paper can be accessed here:

http://www.biomedcentral.com/1471-2458/10/451/prepub

## References

[B1] Hine D The 2009 influenza pandemic: An independent review of the UK response to 2009 influenza pandemic 2010 London: UK Cabinet Office

[B2] Department of Health Pandemic Flu: Managing Demand and Capacity in Healthcare Organisations. (Surge) 2009 London: Department of Health

[B3] Chapman N Wogan M NHS Direct Chief Executives Report 2009 http://www.nhsdirect.nhs.uk/about/minutesofmeetings/~/media/files/boardpapers/june2009/bm_230609_chiefexecutivesreport.ashx

[B4] Health Protection Agency HPA Weekly National Influenza Report 2009 http://www.hpa.org.uk/web/HPAwebFile/HPAweb_C/1243467927021 (Week 22)

[B5] Rubin GJ Dickmann P How to reduce the impact of "low risk patients" following a bioterrorist incident: Lessons from SARS, anthrax and pneumonic plague Biosecurity and Bioterrorism: Biodefense Strategy, Practice, and Science 2010 8 1 37 43 10.1089/bsp.2009.0059 20230231

[B6] Engel CC Locke S Reissman DB DeMartino R Kutz I McDonald M Terrorism, trauma and mass casualty triage: How might we solve the latest mind-body problem? Biosecurity and Bioterrorism: Biodefense Strategy, Practice, and Science 2007 5 155 163 10.1089/bsp.2007.0004 17608601

[B7] Smithson AE Smithson AE, Levy L-A Rethinking the lessons of Tokyo Ataxia: The Chemical and Biological Terrorism Threat 1999 Washington D.C.: Henry L. Stimson Center

[B8] Lister S Britain prepares for 65,000 deaths from swine flu The Times 2009 2009 1

[B9] Important information about swine flu - leaflet 2009 NHS Scotland; NHS Wales; Department of Health, Social Services and Public Safety; NHS

[B10] Mott JA Treadwell TA Hennessy TW Rosenberg PA Wolfe MI Brown CM Call tracking data and the public health response to bioterrorism-related anthrax Emerg Infect Dis 2002 8 10 1088 1092 12396921 10.3201/eid0810.020355 PMC2730299

[B11] Kaydos-Daniels SC Olowokure B Chang HJ Barwick RS Deng JF Lee ML Body temperature monitoring and SARS fever hotline, Taiwan Emerg Infect Dis 2004 10 2 373 376 15030716 10.3201/eid1002.030748 PMC3322900

[B12] O'Cathain A Munro JF Nicholl JP Knowles E How helpful is NHS Direct? Postal survey of callers BMJ 2000 320 1035 10.1136/bmj.320.7241.1035 10764361 PMC27342

[B13] Hohenemser C Kates RW Slovic P The nature of technological hazard Science 1983 220 378 384 10.1126/science.6836279 6836279

[B14] Rubin GJ Page LA Morgan O Pinder RJ Riley P Hatch S Public information needs after the poisoning of Alexander Litvinenko with polonium-210 in London: cross sectional telephone survey and qualitative analysis BMJ 2007 335 1143 10.1136/bmj.39367.455243.BE 17975252 PMC2099556

[B15] Marteau TM Becker H The development of a six-item short-form of the state scale of the Speilberger state-trait anxiety inventory (STAI) Br J Clin Psychol 1992 31 301 306 1393159 10.1111/j.2044-8260.1992.tb00997.x

[B16] Pope C Ziebland S Mays N Pope C, Mays N Analysing qualitative data Qualitative Research in Health Care 2008 Oxford: Blackwell Publishing 63 81

[B17] Petterson JS Perception vs. reality of radiological impact: the Goiania model Nuclear News 1988 31 85 90

[B18] Bleich A Dycian A Koslowsky M Solomon Z Wiener M Psychiatric implications of missile attacks on a civilian population JAMA 1992 268 613 615 10.1001/jama.268.5.613 1629988

[B19] Tham KY An emergency department response to severe acute respiratory syndrome: A prototype response to bioterrorism Ann Emerg Med 2004 43 6 14 10.1016/j.annemergmed.2003.08.005 14707933 PMC7124311

[B20] Chen SY Ma MHM Su CP Chiang WC Ko PCI Lai TI Facing an outbreak of highly transmissible disease: Problems in emergency department response Ann Emerg Med 2004 44 93 5 10.1016/j.annemergmed.2004.01.028 15259174 PMC7124265

[B21] Weinman J Petrie KJ Moss-Morris R Horne R The illness perception questionnaire: A new method for assessing the cognitive representation of illness Psychology and Health 1996 11 431 445 10.1080/08870449608400270

[B22] Figueiras MJ Alves NC Lay perceptions of serious illnesses: An adapted version of the Revised Illness Perception Questionnaire (IPQ-R) for healthy people Psychology and Health 2007 22 2 143 158 10.1080/14768320600774462

[B23] Rubin GJ Amlot R Rogers MB Hall I Leach S Simpson J Public perceptions of and reactions to pneumonic plague Emerg Infect Dis 2009 16 120 122 10.3201/eid1601.081604 PMC2874346 20031056

